# A universal protocol to generate consensus level genome sequences for foot-and-mouth disease virus and other positive-sense polyadenylated RNA viruses using the Illumina MiSeq

**DOI:** 10.1186/1471-2164-15-828

**Published:** 2014-09-30

**Authors:** Grace Logan, Graham L Freimanis, David J King, Begoña Valdazo-González, Katarzyna Bachanek-Bankowska, Nicholas D Sanderson, Nick J Knowles, Donald P King, Eleanor M Cottam

**Affiliations:** The Pirbright Institute, Ash Road, Pirbright, Woking, Surrey GU24 0NF United Kingdom

**Keywords:** Next generation sequencing, Whole genome sequencing, Foot-and-mouth disease virus, Genome, RNA, virus, FMDV

## Abstract

**Background:**

Next-Generation Sequencing (NGS) is revolutionizing molecular epidemiology by providing new approaches to undertake whole genome sequencing (WGS) in diagnostic settings for a variety of human and veterinary pathogens. Previous sequencing protocols have been subject to biases such as those encountered during PCR amplification and cell culture, or are restricted by the need for large quantities of starting material. We describe here a simple and robust methodology for the generation of whole genome sequences on the Illumina MiSeq. This protocol is specific for foot-and-mouth disease virus (FMDV) or other polyadenylated RNA viruses and circumvents both the use of PCR and the requirement for large amounts of initial template.

**Results:**

The protocol was successfully validated using five FMDV positive clinical samples from the 2001 epidemic in the United Kingdom, as well as a panel of representative viruses from all seven serotypes. In addition, this protocol was successfully used to recover 94% of an FMDV genome that had previously been identified as cell culture negative. Genome sequences from three other non-FMDV polyadenylated RNA viruses (EMCV, ERAV, VESV) were also obtained with minor protocol amendments. We calculated that a minimum coverage depth of 22 reads was required to produce an accurate consensus sequence for FMDV O. This was achieved in 5 FMDV/O/UKG isolates and the type O FMDV from the serotype panel with the exception of the 5′ genomic termini and area immediately flanking the poly(C) region.

**Conclusions:**

We have developed a universal WGS method for FMDV and other polyadenylated RNA viruses. This method works successfully from a limited quantity of starting material and eliminates the requirement for genome-specific PCR amplification. This protocol has the potential to generate consensus-level sequences within a routine high-throughput diagnostic environment.

**Electronic supplementary material:**

The online version of this article (doi:10.1186/1471-2164-15-828) contains supplementary material, which is available to authorized users.

## Background

Foot-and-mouth disease (FMD) has been associated with severe productivity losses in cloven-hoofed animals characterised by vesicular lesions of the feet, tongue, snout and teats as well as fever and lameness [1]. The disease has a serious impact upon food security, rural income and significant economic consequences for any country harbouring the virus [2]. An integral part of any viral disease control strategy is the epidemiological tracing of virus transmission together with conventional field investigations. For RNA viruses with high evolutionary rates, this is routinely achieved with the application of molecular and phylogenetic methods [3–5] one example being the global tracing of foot-and-mouth disease virus (FMDV) [6]. Next-generation sequencing platforms offer much promise as rapid, cost-effective, and high-throughput methods for the generation of viral genome sequences. Recovering whole genome consensus level sequences of viruses provides important information for outbreak epidemiology and pathogen identification [7–10].

The positive-sense single-stranded RNA genome of FMDV is comprised of a single long open reading frame. This encodes a polyprotein which is flanked by 5′ and 3′ untranslated regions of approximately 1200 nt and 95 nt, respectively, terminating in a poly (A) tail. The 5′ UTR contains highly structured RNA which is involved in both replication and translation. Approximately 300–370 nt from the 5′ end of the genome lies a homopolymeric cytidylic acid [poly(C)] tract of ~100-170 nt [11]. The genome sequence upstream of the poly(C) tract is known as the S fragment and that downstream as the L fragment.

Previously, tracing and monitoring of the trans-boundary movements of FMDV has been successfully achieved using consensus sequences of the VP1 region [12–14]. However, over shorter epidemic time scales, where viral populations have not substantially diverged, VP1 sequencing cannot provide the required resolution to discriminate between viruses in field samples collected from neighbouring farms within outbreak clusters. At this scale, WGS at the consensus level has proven to be a powerful tool for the reconstruction of transmission trees [15].

Previous strategies for viral WGS include PCR and Sanger sequencing methods or microarray approaches [15, 16]. Commonly, these processes have limited throughput and are both resource and labour-intensive with biased outputs that may not reflect the true diversity within samples [17, 18]. Furthermore, such methodologies have been subject to errors incumbent within the nature of the protocol i.e. those protocols reliant upon DNA amplification generate biased datasets from which it is difficult to make firm conclusions [19]. Such strategies have also been dependent upon *a priori* knowledge of virus sequences for primer design and are limited by potential inter and intra-sample sequence variation [20].

This study describes the optimisation of a robust, high-throughput protocol for WGS of all seven serotypes of FMDV excluding the 5′ genomic termini and poly(C) tract. It does not use PCR amplification prior to the sequencing steps and overcomes the requirement for large starting quantities of template nucleic acid, which has previously limited the suitability of some NGS technologies for processing viral field isolates [21–23]. This protocol, with minor changes, was also applied to other polyadenylated RNA viruses.

## Results

### Protocol accuracy: calculation of minimum coverage required for accurate consensus

**Figure 1:**
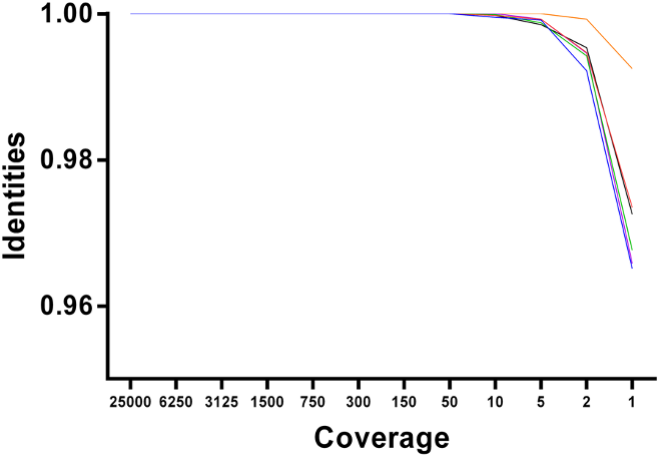
**Read coverage required to obtain an accurate consensus sequence.** The consensus sequence resulting from varying levels of coverage was assessed for accuracy. Isolates O/UKG/1450/2001 (blue), O/UKG/1558/2001 (green), O/UKG/1734/2001 (purple), O/UKG/4998/2001 (orange) and O/UKG/14597/2001 (red) alongside the type O exemplar from the serotype panel (black) were analysed. Points on the graph represent a comparison of the identities (scored on the y axis) of a consensus made with total reads and a consensus made with limited read coverage (detailed on the x axis). On average, an identity score of one was maintained up to (and including) a coverage limit of 22 reads. Below this level of coverage, the accuracy of the identities of the compared consensus sequences decreased i.e. consensus sequences made with a depth of 22x reads were identical to the consensus. Sequences created with less than 22x coverage depth were not identical, and therefore considered less accurate.

Next-generation sequencing analysis provided large numbers of short read sequences that were assembled and aligned in order to determine a consensus sequence. To define how much redundancy was required for accurate reconstruction of consensus level sequences, we determined the minimum read coverage required to obtain a robust consensus from the protocol described. Analysis was completed on all FMDV type O samples with sufficient coverage (Figure 1). From this a mean was calculated showing a minimum coverage of 22 reads was required to obtain an accurate consensus sequence in this instance.

### Analytical sensitivity of WGS protocol: consensus sequence was obtained to 1 × 10^7^ virus genome copies

The protocol workflow (See Materials and Methods) was optimised and tested using a single FMDV O/UKG/35/2001 isolate. Initially, the sensitivity of the protocol in the presence of gDNA (i.e. no rDNase1 treatment) was tested against viral dilutions spanning 1 × 10^8^, 1 × 10^7^ and 1 × 10^6^ RNA copies/μl. The total number of Illumina reads in all five samples ranged between 2.5 × 10^6^ and 1.2 × 10^6^ (Table 1). Consensus genome sequences (8176 nucleotides in length) were created from alignments of these reads at each dilution. A decreasing percentage of viral reads correlated with decreasing viral load (17.94%, 14.41%, 1.83%, 0.05% and 0.01% respectively). Consensus sequences were found to be identical in all cases both between individual samples and the reference sequence (data not shown). For this isolate, whole genome sequence was attained (excluding the 5′ termini) for 1 × 10^8^ and 1 × 10^7^ genomes copies/μl, however, below this level, coverage was incomplete. Coverage was increased in regions adjacent to primer binding sites and was lowest in the S-fragment (genome positions nt 1–376), notably in regions immediately adjacent to the poly(C) tract. The 3’ genomic termini were obtained in the cell culture neat virus sample (1 × 10^8^ copies/μl) with only 2 bases missing at the 5′ termini. In order to gain accurate consensus our analysis shows that for type O we needed a minimum viral read depth of 22. By this criterion accurate consensus sequences were generated for >98.1% of the genome, down to 1 × 10^7^ copies/μl. Below this threshold (i.e. <1 × 10^7^ copies/μl) we observed a rapid drop-off in the coverage depth of genome sequences with average coverage across the genome dropping from 639 (1 × 10^7^) to 18 (1 × 10^6^) (Table 1). Furthermore both genomic termini, notably the 5′ end, were also lost with decreasing viral load.

**Table 1:** **Library complexity of all samples run whilst optimising the protocol for whole genome sequencing**

Sample ID	Serotype	Dnase treatment	Viral load (cp/μl)	Total no. reads	Total viral reads	Percentage viral reads	Mean coverage across genome	Percentage consensus > depth 22
**UKG/35/2001**	FMDV-O	N	4.47 × 10^8^	1.21 × 10^6^	2.17 × 10^5^	17.94	3965	99.28
**UKG/35/2001**	FMDV-O	N	1.65 × 10^8^	1.77 × 10^6^	2.55 × 10^5^	14.41	4641	99.3
**UKG/35/2001**	FMDV-O	N	3.98 × 10^7^	1.92 × 10^6^	3.51 × 10^4^	1.83	639	98.12
**UKG/35/2001**	FMDV-O	N	7.94 × 10^6^	2.08 × 10^6^	1 × 10^3^	0.05	18	38.35
**UKG/35/2001**	FMDV-O	N	1.35 × 10^6^	2.47 × 10^6^	1.75 × 102	0.01	3	0
**UKG/35/2001**	FMDV-O	Y	4.47 × 10^8^	4.63 × 10^5^	1.19 × 10^5^	25.83	2178	99.36
**UKG/35/2001**	FMDV-O	Y	1.65 × 10^8^	1.76 × 10^5^	4.11 × 10^4^	23.37	743	98.29
**UKG/35/2001**	FMDV-O	Y	3.98 × 10^7^	3.29 × 10^5^	8.29 × 10^3^	2.52	149	93.71
**UKG/35/2001**	FMDV-O	Y	7.94 × 10^6^	4.62 × 10^5^	1.07 × 10^3^	0.23	19	35.71
**UKG/35/2001**	FMDV-O	Y	1.35 × 10^6^	3.73 × 10^5^	1.11 × 10^2^	0.03	2	0
**UKG/1734/2001**	FMDV-O	Y	2.89 × 10^8^	5.14 × 10^5^	4.12 × 10^5^	80.12	6961	99.46
**UKG/1450/2001**	FMDV-O	Y	4.95 × 10^8^	1.23 × 10^6^	1.10 × 10^6^	88.97	18362	99.72
**UKG/14597/2001**	FMDV-O	Y	1.77 × 10^8^	2.94 × 10^5^	2.03 × 10^5^	69.02	3557	97.67
**UKG/1558/2001**	FMDV-O	Y	4.39 × 10^8^	6.11 × 10^5^	5.27 × 10^5^	86.29	9391	99.68
**UKG/4998/2001**	FMDV-O	Y	1.01 × 10^7^	2.97 × 10^4^	2.01 × 10^4^	67.49	352	80.55
**TUR/11/2013**	FMDV-O	Y	2.22 × 10^9^	1.29 × 10^6^	8.22 × 10^5^	63.92	14848	99.57
**TUR/12/2013**	FMDV-A	Y	7.06 × 10^8^	1.18 × 10^6^	5.51 × 10^5^	46.49	10011	-
**KEN/1/2004**	FMDV-C	Y	4.41 × 10^8^	1.17 × 10^6^	4.61 × 10^5^	39.45	8049	-
**TUR/13/2013**	FMDV-Asia 1	Y	2.03 × 10^9^	1.69 × 10^6^	9.04 × 10^5^	53.61	10241	-
**TAN/22/2012**	FMDV-SAT 1	Y	1.14 × 10^9^	1.43 × 10^6^	7.26 × 10^5^	50.9	13185	-
**TAN/5/2012**	FMDV-SAT 2	Y	1.35 × 10^9^	1.18 × 10^6^	5.35 × 10^5^	45.48	9724	-
**ZIM/6/91**	FMDV-SAT 3	Y	1.47 × 10^9^	2.70 × 10^6^	1.36 × 10^5^	50.21	2453	-
**VR-129B**	EMCV-1	Y	-	2.63 × 10^6^	2.12 × 10^6^	80.34	31208	-
**D1305-03**	ERAV-1	Y	-	3.78 × 10^4^	2.68 × 10^4^	70.98	409	-
**B1-34**	VESV-B34	Y	-	4.77 × 10^5^	6.84 × 10^4^	14.34	1112	-
**ISR/2/2013**	FMDV-O	Y	4.50 × 10^6^	16 × 10^4^	1.05 × 10^3^	6.53	18	-

N = no; Y = yes; cp = copies.

Different factors of library complexity including total number of reads, number of viral reads, coverage and mean coverage depth across the genome (percentage consensus depth indicates areas in which depth is over 22).

### gDNA depletion increases proportion of reads attributed to virus genome

We investigated the impact of genomic DNA (gDNA) depletion by rDNase1 treatment upon the final library complexity. Removal of gDNA was confirmed by Qubit measurement before and after treatment (data not shown). Although the majority of DNA in the sample was eliminated it should be noted that some residual DNA remained in the sample. Samples that had not been subjected to rDNase1 treatment contained increased total number of reads, compared to samples that had been treated with rDNase1 (average: 1.9 × 10^6^ vs. 3.8 × 10^5^ reads, respectively). However, a higher percentage of reads aligned with the reference template for gDNA depleted samples compared to untreated samples (Table 1).

### Validation of protocol on field samples of FMDV and reproducibility

**Figure 2:**
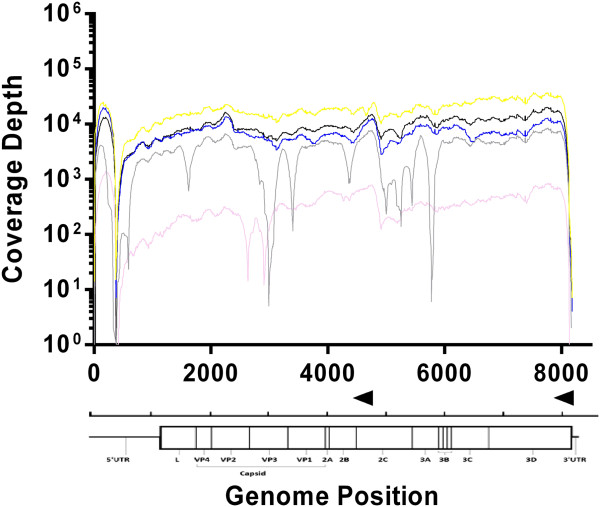
**Application of protocol to field isolates from 2001.** Coverage of between 1000–10,000x was achieved for 4/6 UKG 2011 isolates (O/UKG/1450/2001 (blue), O/UKG/1558/2001 (green), O/UKG/1734/2001 (purple) and O/UKG/14597/2001 (red)) with a drop in coverage at the poly(C) tract (~375 bp position). O/UKG/4998/2001 (orange) showed lower coverage of between 10-100x. Primer locations are shown as black arrowheads above the genome illustration.

Five field samples submitted to the UK FMD National Reference Laboratory (Pirbright, UK) during the UK 2001 outbreak were tested using the sequencing protocol for UKG specific viruses as described above. Virus load in all samples was quantified by real-time RT-qPCR (Table 1). Four of five samples (O/UKG/1450/2001, O/UKG/1558/2001, O/UKG/1734/2001 and O/UKG/14597/2001) contained between 1.8 × 10^8^ – 5.0 × 10^8^ copies/μl. The remaining sample (O/UKG/4998/2001) was of lower viral loads with 1.01 × 10^7^ copies/μl, respectively. The number of viral reads per sample varied between 1 × 10^6^ (sample O/UKG/1450/2001) and 1 × 10^4^ (O/UKG/4998/2001), potentially reflecting differences in viral load. Reads were trimmed and aligned to a reference sequence FMDV O/UKG/35/2001 (AJ539141). All samples exhibited increased coverage at primer specific sites (Figure 2) and decreased coverage at the sites adjacent to the FMDV poly(C) tract and at the 5′ termini of the S fragment. Samples with viral load >1 × 10^8^ copies/μl exhibited >69% of reads aligning to the reference template. The sample with the lowest viral load, O/UKG/4998/2001, resulted in 67.5% of reads aligning to the template. Complete genome sequences (excluding genomic termini) were obtained for all samples. Isolate O/UKG/1450/2001, which exhibited the highest viral load and total numbers of reads, generated a coverage depth >22 across 99.72% of the genome.

For the five samples that generated a whole genome sequence, the coverage across the L fragment was even, peaking in regions of reverse transcription primer binding (Figure 2). All genome sequences have been submitted to GenBank (KM257061-KM257065). To evaluate reproducibility, one isolate (O/UKG/35/2001) was sequenced 15 separate times. Analysis was completed on each of these 15 repeats and no changes in the consensus sequence produced were observed.

### Application to cell culture negative FMDV

A diagnostic virus sample O/ISR/2/2013, submitted to the WRLFMD in 2013, was sequenced using the whole genome sequencing protocol. The virus could not be isolated in cell culture, but FMDV RNA was detected with diagnostic real-time reverse transcription-quantitative PCR (RT-qPCR) and quantified as 4.5 × 10^6^ copies/μl (Table 1). The majority of the genome sequence was generated [(94.10%), with an average coverage depth of 18] with several gaps evident across the genome length (Additional file 1: Figure S1).

### Pan-FMDV application of WGS protocol

**Figure 3:**
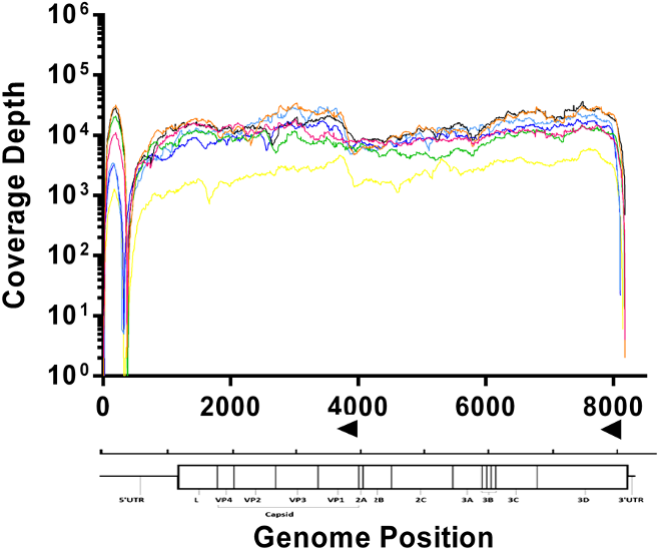
**Genome coverage profiles for FMDV serotype panel.** Sequence data coverage at each position along the genome is shown for serotype O (black), A (pink), Asia 1 (orange), C (green), SAT 1 (light blue), SAT 2 (blue), and SAT 3 (yellow). The majority of the coverage is above 1000x. In all viruses tested, a poly(C) tract within the FMDV genome at ~375 bp was associated in a reduction in coverage. The coverage depth observed for SAT 3 was lower than other serotypes. Primer locations are shown as black arrowheads above the genome illustration.

After validation with FMDV UKG field samples the protocol was used to determine whole genome sequences for a panel of RNA viruses representing each of the seven FMDV serotypes (Figure 3). In order to optimise the protocol we replaced the type O specific primer ‘4926R’ with a pan-FMDV primer ‘NK-72’ designed to bind a region conserved between all seven FMDV serotypes (Table 2). The panel had a viral load >1 × 10^8^ copies/μl. *De*-*novo* assemblies were completed to provide a consensus against which all reads were aligned. All viruses gave similar depth of coverage (approx. 1 × 10^4^) and exhibited comparable library complexity with the exception of SAT 3 whose depth of coverage was reduced (average coverage: 1 × 10^3^) (Table 1). The 5′ genomic termini was also missing from all panel viruses ranging from 9 bases of A and Asia1 to 15, 17, 22 and 27 for SAT 2, SAT 1, SAT 3 and O respectively (accession numbers KM268895-901).

**Table 2:** **Primers and probes used in quantitation and WGS of FMDV and other RNA viruses**

	Primer name	Primer sequence
**RT**-**qPCR**	Callahan 3DF [24]	ACT GGG TTT TAC AAA CCT GTG A
	Callahan 3DR [24]	GCG AGT CCT GCC ACG GA
	Callahan 3DP [24]	TCC TTT GCA CGC CGT GGG AC
**First**-**strand synthesis**	UKFMD Rev 6 [25]	GGC GGC CGC TTT TTT TTT TTT TTT
	NK72 [26]	GAA GGG CCC AGG GTT GGA CTC
	UKFMD UKG 4926R	AAG TCC TTC CCG TCG GGG T
	EMC-2B65R [27]	TCG GCA GTA GGG TTT GAG
	ERAV-2A22R [28]	GGG TTG CTC TCA ACA TCT CCA GCC AAT TT
	Vesi-3D1R	CKN GTN GGY TTN ARN CC
	Vesi-3D2R	TAN CAN CCR TCR TCN CCR TAN GT

International Union of Pure and Applied Chemistry (IUPAC) nucleotide ambiguity codes:

N: G or T or A or C; K: G or T; Y: T or C; R: G or A.

### Application to non-FMDV RNA viruses

**Figure 4:**
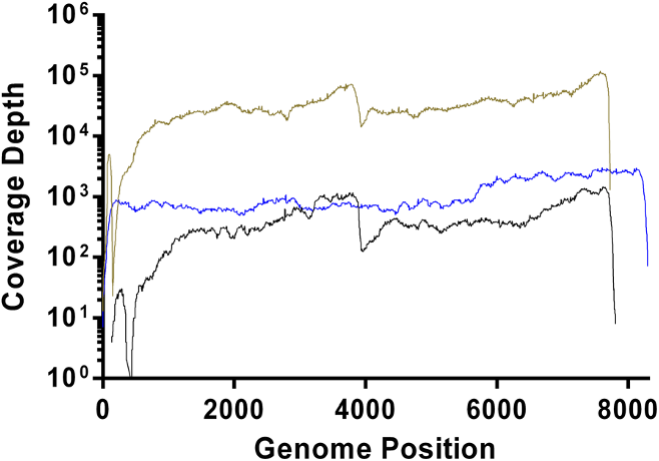
**Genome coverage profiles for three non**-**FMDV panel of viruses.** Coverage of 10,000 was achieved for the majority of the EMCV-1 genome (olive). Peaks in coverage can be observed at the location of sequence specific primers used in the RT reaction (~4000 bp and ~8000 bp). A dip in coverage was evident at the poly(C) tract. The ERAV-1 genome showed between 10x and 100x coverage with visible peaks in coverage at the specific primer sites (~4000 bp and ~8000 bp) (black). Approximately 100x coverage of the majority of the VESV-B34 genome was achieved (blue).

In order to demonstrate the suitability of this method to attain whole genome sequence from other poly(A) tailed RNA viruses, we tested the protocol upon three different viruses including encephalomyocarditis virus 1 (EMCV-1) equine rhinitis A virus 1 (ERAV-1) and vesicular exanthema of swine virus B34 (VESV-B34) (Figure 4). For all three viruses, first-strand cDNA synthesis was performed using the 3′ oligo-dT primer ‘Rev 6’ and sequence-specific primers replacing the pan-FMDV specific NK72 (Table 2). The complete genome sequence, apart from the poly(C) tract was determined for EMCV-1 ATCC VR-129B (KM269482). The complete genome sequence, apart from 100+ nt at the 5′ end of the genome was determined for ERAV-1 D1305-03 (KM269483). Similarly, the majority of the calicivirus VESV-B34 genome was determined apart from six nt at the 5′ end of the genome (KM269481).

## Discussion

We have described a novel sample preparation method incorporating minimal amplification for the accurate sequencing of RNA viruses to a consensus level, using an Illumina MiSeq. This protocol is an affordable and reproducible method to generate whole genome sequences for FMDV and other RNA viruses, which could be adapted to routine high-throughput diagnostic laboratory workflows. The protocol was validated using FMDV type O (Figure 2) and shown to be applicable to all other serotypes of FMDV (types A, C, Asia 1, SAT 1, SAT 2 and SAT 3) (Figure 3) as well as other picornaviruses (EMCV-1 and ERAV-1) and a calicivirus (VESV-B34) (Figure 4).

We have shown that the protocol is able to produce whole genome sequences from samples with viral loads as low as 1 × 10^7^ virus RNA copies per μl. Further validation was performed with five samples submitted during the UK 2001 FMDV outbreak. The generation of five genomes from these samples, without PCR amplification or virus culture, demonstrated the potential for this method to investigate larger outbreak sample sets in a high-throughput, diagnostic setting, such as the UK 2001 FMDV outbreak.

PCR processes have previously been shown to be error prone [4] and thus eradication of this step has the opportunity to improve the quality of the data. Our protocol differs from previous studies in the literature through inclusion of sequence specific primers, as opposed to random priming at the first strand cDNA stage [29, 30]. This decision was made with the intent of maximising coverage, across the whole genome, specifically for FMDV; although it is possible that primer induced bias could be introduced into sequences through use of sequence specific primers.

We have also demonstrated the effectiveness of adapting this method for WGS of other RNA viruses (Figure 4). We foresee this protocol being practicable for unknown positive sense polyadenylated viruses through use of random primers and, where appropriate, an oligo-dT primer.

The specificity previously provided by PCR has been replaced with reduction of host DNA and the optional use of specific primers in the reverse transcription reaction. Instead of enriching viral RNA we depleted host genomic DNA. We did not target ribosomal RNA in order to keep reagent costs low thus maintaining the suitability of the protocol for ‘high-throughput’ sample processing. The method described here was capable of generating whole genome sequences of FMDV field isolates with a coverage depth of up to 1 × 10^4^ (data not shown) that was considered sufficient for the study of minority variants [24], with only a minimal amount of PCR at the library preparation stage. This PCR amplification involved 10 cycles of amplification by a hi-fidelity DNA polymerase, thereby posing minimal risk to biasing the final sequence data [31].

It was evident that in genome sequences generated using this protocol the genomic termini and poly(C) tract exhibited lower coverage depths. The 5′ genomic termini were always under-represented within the genomes. This was particularly evident in samples of decreased viral load suggesting that increasing the input RNA of such samples could improve this coverage. Additionally homopolymeric regions, such as the long poly(C) tract of FMDV, have been demonstrated here to cause significant decreases in coverage. With Sanger sequencing, large parts of the genome are often missing or primer derived. For example, twenty seven to fifty nucleotides of the full genome sequences obtained by Sanger sequencing described by Valdazo-Gonzalez et al., [15, 32–34] were primer derived (from the forward and reverse primers to amplify 5’ and 3’ termini of both the S and the L fragment) and thus the method described here offers a notable improvement on the resolution of these regions. As previously stated, a minimum read depth of coverage required to create an accurate consensus for a type O sequence was on average 22 (Figure 1). Even after implementation of this criterion, consensus sequences were generated with a depth of >22, at more than 80.6% genome positions. This was observed in the 5 UKG field isolates tested and >99.6% for type O virus tested as part of the panel of serotypes (Table 1).

Such advances in WGS will likely impact fields such as virus evolution, diagnosis, and generation of high/low pathogenicity variants. We have already shown this method can be advantageous in a diagnostic setting with the successful sequencing of 94.1% of the genome of a culture negative field isolate. FMDV reads were successfully identified although the resulting profile exhibits several gaps in the genome sequence suggesting that the RNA was in fact degraded - an observation potentially explaining the inability for this virus to grow successfully in cell culture. For this protocol to be fully functional within a diagnostic environment, it remains to be confirmed whether it is able to correctly identify all viruses or serotypes within mixed samples.

## Conclusion

This paper outlines the development of a high-throughput protocol for the generation of whole genome sequences of all seven serotypes of FMDV. With minimal changes applied to priming in the first strand synthesis stage such a strategy can be tailored to other RNA viruses. The application of NGS to virology will prove invaluable to the fields of molecular epidemiology and phylogenetic outbreak tracing. This paper describes a fast, robust and affordable protocol, which is essential to realise this potential.

## Methods

### Virus specimens

The protocol was initially developed and validated using an FMDV field isolate (O/UKG/35/2001) submitted to the FAO World Reference Laboratory for FMD (WRLFMD, Pirbright, UK) during the 2001 FMD outbreak in the United Kingdom. It was further validated with a panel of other samples originating from this outbreak as well as with a panel of viruses representing all FMDV serotypes. The protocol was also validated with other representative polyadenylated RNA viruses. The details of all viruses used in the study are described in Table 3. Where appropriate, viruses were cultured for one replication cycle in bovine thyroid cells (BTy) as described previously [35]. Dilutions between 1 × 10^8^ to 1 × 10^6^ viral copies/μl of O/UKG/35/2001 were made with viral cell culture supernatant in virus negative suspensions of bovine epithelium to mimic real clinical samples with different viral loads.

**Table 3:** **Viruses used in development and validation of the non**-**amplification protocol**

Family	Genus	Species	Serotype	Isolate	Passage history (Cell type/Passage number)
*Picornaviridae*	*Aphthovirus*	*Foot*-*and*-*mouth disease virus*	O	UKG/1734/2001	10% epith. susp.
				UKG/1450/2001	10% epith. susp.
				UKG/14597/2001	10% epith. susp.
				UKG/1558/2001	10% epith. susp.
				UKG/4998/2001	10% epith. susp.
				UKG/1485/2001	10% epith. susp.
				UKG/35/2001	10% epith. susp.
				TUR/11/2013	BTy2
			A	TUR/12/2013	BTy2
			C	KEN/1/2004	BTy2
			Asia 1	TUR/13/2013	BTy2
			SAT 1	TAN/22/2012	BTy2
			SAT 2	TAN/5/2012	BTy2
			SAT 3	ZIM/6/91	BTy2
		*Equine rhinitis A virus*	1	D1305-03, dromedary, Dubai, 2003	Vero2
	*Cardiovirus*	*Encephalomyocarditis virus*	1	VR-129B, chimpanzee, Florida, 1944	BHK3
*Caliciviridae*	*Vesivirus*	*Vesicular exanthema of swine virus*	B34	B1-34, pig, California, 1934	PK5, IB-RS5

***BTy***: Primary Bovine Thyroid; ***PK***: Pig kidney epithelial cells; ***BHK***: Baby Hamster Kidney; ***IB***-***RS***: instituto Brazilia Renal Swine; Numbers denote passage number.

### RNA extraction & FMDV-specific RT-qPCR

Total RNA was extracted from 460 μl of cell culture virus isolate or original suspension [consisting of 10% tissue suspensions generated in M25 phosphate buffer (35 mM Na_2_HPO_4_.2H_2_O; 5.7 mM KH_2_PO_4_; pH 7.6; made in-house)] using RNeasy MiniKit (Qiagen) according to manufacturer’s instructions. Total RNA was eluted in 50 μl of nuclease-free water and quantified using the Qubit RNA High Sensitivity (HS) Assay Kit (Life Technologies). FMDV-specific RNA was detected using an FMDV-specific real-time RT-qPCR as described previously (Table 2) [24] and quantified using an RNA standard derived from O/UKG/35/2001.

### gDNA depletion

Genomic DNA (gDNA) was depleted from extracted total RNA samples through the activity of rDNase1 using the DNA-free DNAse kit (Life Technologies). Briefly, 10 μg of extracted nucleic acid in a 50 μl volume was combined with 5 μl of DNase Buffer and 1 μl of rRDNase1 (2 U), and incubated at 37°C for 30 min. Inactivation agent was added as per manufacturer’s protocol and the sample was incubated for a further 2 min at room temperature with periodic mixing. The samples were then centrifuged at 17,000 *xg* for 2 min and the DNAse-treated supernatant was retained for subsequent processing.

### cDNA synthesis

First-strand cDNA synthesis (reverse transcription) was performed using Superscript III First-Strand Synthesis System (Life Technologies) according to the manufacturer’s protocol. Briefly, 10 μl of DNase-treated total RNA was combined with oligonucleotide primers (Rev6 (2 μM), NK72 (2 μM) or FMDV-4926R (2 μM)) depending on the application of the protocol, random hexamers (50 ng/μl: Life technologies), dNTPS (10 mM: Life Technologies) and nuclease-free water (Life Technologies) (Table 2). Reactions were incubated at 65°C for 5 min and cooled on ice for 5 min. A second reagent mix was added containing SuperScript III enzyme (200 U: Life Technologies), RNaseOUT (40 U: Life Technologies), 0.1 M dTT (life Technologies) and 25 mM MgCl_2_, before incubating at 50°C for 50 min. A final incubation with RNase H (2 U: Life Technologies) was then performed at 37°C for 20 min.

Second-strand synthesis was performed using NEB Second Strand Synthesis kit (NEB) as per manufacturer’s instructions using 20 μl of cDNA. The resulting dsDNA was purified using Illustra GFX DNA/gel clean-up kit (GE) as per manufacturer’s instructions and samples eluted in 30 μl of nuclease-free water. Double-stranded cDNA samples were then quantified using the Qubit dsDNA High Sensitivity (HS) Qubit kit (Life Technologies) after which samples were adjusted to 0.2 ng/μl using nuclease-free water where appropriate prior to library preparation.

### Illumina library preparation

One nanogram of each dsDNA sample was used to prepare sequencing libraries using the Nextera XT DNA Sample Preparation Kit (Illumina) according to manufacturer’s instructions. Libraries were sequenced on a MiSeq using 300 cycle version 2 reagent cartridges (Illumina) to produce paired end reads of approximately 150 bp each.

### Sequence data analysis

Consensus sequences were attained using a complete published sequence as a template or, where a closely related template was not available, a *de novo* assembly. Sequence read quality was monitored with FastQC [36] prior to Sickle [37] trimming all bases with a q score of <30. For *de novo* trimmed Fastq files were processed using Velvet v1.2.10 [38] with an optimum Kmer length determined by Velvet-Optimiser. A minimum contig length of 1000 was included in L fragment analysis. A BLAST search with the contigs confirmed viral origin [39]. Final contig assemblies were completed manually in BioEdit [40]. Alignments between MiSeq data and appropriate reference genome (from publication or *de novo* assembly) were completed using Bowtie2.1.0 [41] and SAM/BAM processing carried out using Samtools [42]. Alignments were visually checked using Tablet [43]. Coverage data and graphs were generated using Bedtools [44] with final graphical output produced using Prism v6 (GraphPad).

### Sequence data deposition

All genome sequences produced in this study were submitted to NCBI GenBank under the following accession numbers.

### UK2001 FMDV field isolates

O/UKG/1450/2001 [KM257061], O/UKG/1558/2001 [KM257062], O/UKG/1734/2001 [KM257063], O/UKG/14597/2001 [KM257065] and O/UKG/4998/2001 [KM257064].

### Different FMDV serotypes isolates

O/TUR/12/2013 [KM268895], A/TUR/11/2013 [KM268896], C/KEN/1/2004 [KM268897], Asia1/TUR/13/2013 [KM268898], SAT1/TAN/22/2012 [KM268899], SAT2/TAN/5/2012 [KM268900] and SAT3/ZIM/6/91 [KM268901].

### Non-FMDV viruses

VESV-B34 [KM269481], EMCV-1 VR-129B [KM269482] and ERAV-1 D1305-03 [KM269483].

### Read coverage required to obtain an accurate consensus sequence

A sorted alignment file (.sam) of FMDV O/UKG/35/2001 was generated using Bowtie2.1.0 [45] and Samtools [42]. A bespoke python script that truncated the samtools mpileup output format (available upon request) was used to simulate files with varying levels of coverage. A consensus sequence was generated from each of these files using mpileup (Samtools). The consensus sequences created were compared in BioEdit [40] and their sequence identities recorded. This was completed for all FMDV type O isolates with a sufficient number of reads and the mean was calculated.

### Ethics

Our animal use protocols conform to the Animal Research: Reporting In Vivo Experiments (ARRIVE) guidelines [46] for reporting animal studies. All samples were collected with the informed institutional and client consent under the highest standards of veterinary care.

## Authors’ information

Grace Logan and Graham L. Freimanis are joint first authors.

## Electronic supplementary material

Additional file 1: Figure S1: A. Genome coverage profile for FMDV/O/ISR/2/2013. The Israel 2013 isolate of FMDV O was negative when tested in cell culture in IB-RS-2 and BTy cells. This protocol provided coverage of above 10x for the majority of the genome although full genome consensus was not acquired. The expected dip in coverage at the poly(C) was observed. Primer locations are shown as black arrowheads above the genome illustration. (PNG 721 KB)

## Abbreviations

NGS: Next generation sequencing
WGS: Whole genome sequencing
FMDV: Foot-and-mouth disease virus
FMD: Foot-and-mouth disease
PCR (RT-qPCR): Reverse transcription-quantitative
EMCV-1: Encephalomyocarditis virus 1
ERAV-1: Equine rhinitis A virus 1
VESV-B34: Vesicular exanthema of swine virus B34
WRLFMD: World Reference Laboratory for FMD
UTR: Untranslated region
VP1: Viral protein 1
gDNA: Genomic DNA
BTy: Bovine thyroid cells
PK: Pig kidney epithelial cells
BHK: Baby hamster kidney cells.
