# Supplementary figures and images for: A universal protocol to generate consensus level genome sequences for foot-and-mouth disease virus and other positive-sense polyadenylated RNA viruses using the Illumina MiSeq
Source: BMC Genomics. 2014 Sep 30;15(1):828. doi: 10.1186/1471-2164-15-828 (PMC4247156; doi:10.1186/1471-2164-15-828)

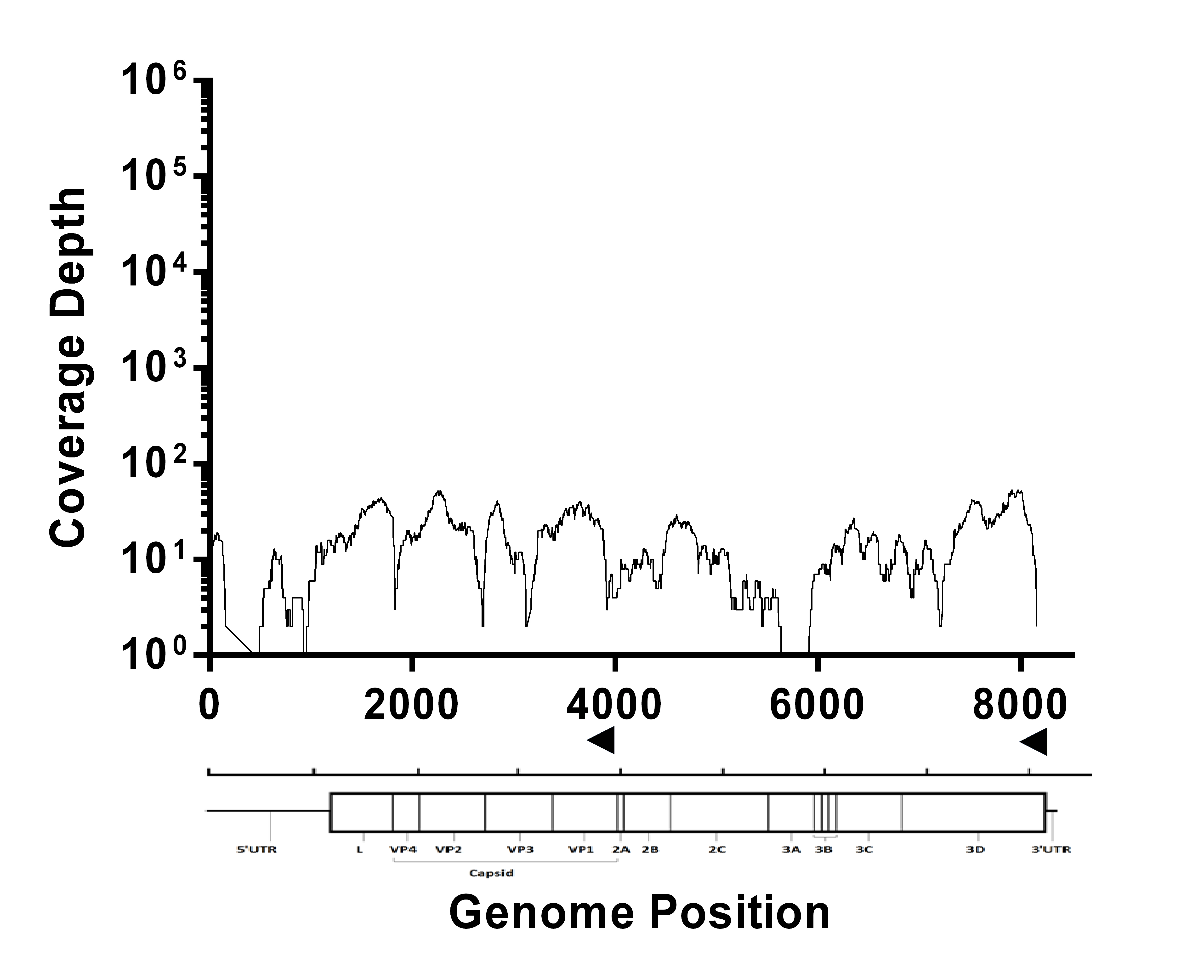

Supplement: Supplementary file 1 — Additional file 1: Figure S1: A. Genome coverage profile for FMDV/O/ISR/2/2013. The Israel 2013 isolate of FMDV O was negative when tested in cell culture in IB-RS-2 and BTy cells. This protocol provided coverage of above 10x for the majority of the genome although full genome consensus was not acquired. The expected dip in coverage at the poly(C) was observed. Primer locations are shown as black arrowheads above the genome illustration. (PNG 721 KB) [file 12864_2014_6688_MOESM1_ESM.png]
